# Platelet Reactivity in the Exacerbation of Psoriasis

**DOI:** 10.3390/jcm13040965

**Published:** 2024-02-08

**Authors:** Piotr Adamski, Urszula Adamska, Katarzyna Buszko, Joanna Sikora, Rafał Czajkowski

**Affiliations:** 1 Department of Cardiology and Internal Medicine, Collegium Medicum, Nicolaus Copernicus University, 85-094 Bydgoszcz, Poland; 2 Department of Dermatology and Venerology, Collegium Medicum, Nicolaus Copernicus University, 85-094 Bydgoszcz, Poland; urszula.adamska@cm.umk.pl (U.A.); r.czajkowski@cm.umk.pl (R.C.); 3 Department of Theoretical Foundations of Biomedical Science and Medical Informatics, Collegium Medicum, Nicolaus Copernicus University, 87-067 Bydgoszcz, Poland; buszko@cm.umk.pl; 4 Research and Education Unit for Experimental Biotechnology, Department of Transplantology and General Surgery, Collegium Medicum, Nicolaus Copernicus University, 85-094 Bydgoszcz, Poland; joanna.sikora@cm.umk.pl

**Keywords:** light transmission aggregometry, platelet reactivity, psoriasis

## Abstract

**Background:** Psoriasis is a chronic, inflammatory, immune-mediated disease with a specific cutaneous presentation. Increased platelet aggregation has been observed in patients with extensive psoriatic lesions. The aim of this study was to evaluate the clinical factors affecting platelet reactivity in patients with an exacerbation of psoriasis. **Methods:** This was a prospective, single-center, observational study, enrolling patients hospitalized for an aggravation of psoriasis. Enrolled patients underwent single platelet function testing with light transmission aggregometry on the first morning of hospitalization. **Results:** 120 patients were enrolled in the study. Of the compared subgroups, women had higher maximal platelet aggregation (MPA) than men (77% vs. 72%; *p* = 0.03), and those with BMIs < 25 kg/m^2^ showed higher platelet reactivity compared to subjects with BMIs ≥ 25 kg/m^2^ (75% vs. 73%; *p* = 0.02). There was a positive correlation between MPA and platelet count (r = 0.27; *p* < 0.01), as well as C-reactive protein concentration (r = 0.20; *p* = 0.03), while a negative correlation was observed with total cholesterol (r = −0.24; *p* = 0.01) and triglycerides (r = −0.30; *p* < 0.01). A two-step analysis based on multidimensional models with random effects revealed that every increase in the platelet count by 10^3^/μL led to an increase in MPA by 0.07% (R^2^ = 0.07; *p* < 0.01), and an increase in triglycerides’ concentration by 1 mg/dL was related to a reduction in MPA by 0.05% (R^2^ = 0.07; *p* < 0.01). **Conclusions:** The increased platelet reactivity observed in patients with psoriasis appears to be multifactorial and related to several clinical and laboratory features. Further research is warranted to put these findings into a clinical perspective.

## 1. Introduction

Psoriasis is a chronic, inflammatory, immune-mediated disease with a specific cutaneous presentation. The prevalence of psoriasis varies geographically, and ranges from 0.5% in Asia to 8% in Scandinavia, with an estimated 125 million people affected worldwide [1,2]. The four main variants of psoriatic disease include plaque psoriasis, guttate psoriasis, erythrodermic psoriasis, and pustular psoriasis, the first one being the most common type. Clinical features vary depending on the type of the disease, but erythema, thickening, and scale are usually present [2].

The pathophysiology of psoriasis is complex and not entirely understood. Psoriasis appears to have a multifactorial background, including genetic, immunological, and environmental determinants [2,3,4,5]. Nevertheless, available data suggest that excessive activation of the adaptive immune system is central to the pathogenesis of psoriasis [6]. Multiple cell types, including activated myeloid dendritic cells or different subpopulations of T cells, participate in a cytokine-dependent pathway that activates intracellular signal transduction in keratinocytes leading to the gene transcription of cytokines and chemokines. Eventually, this results in an inflammatory cascade that leads to manifestations of psoriasis, but it also plays a crucial role in the breakdown of atherosclerotic plaques [2,6,7]. Accordingly, chronic inflammation is a recognized risk factor of cardiovascular disease [8]. The severity of coronary artery disease in patients with psoriasis appears to be comparable to that seen in patients with diabetes or hyperlipidemia [9,10]. Moreover, patients with severe psoriasis are at 42% greater risk of adverse major cardiovascular events, such as cardiovascular death, myocardial infarction, or cerebrovascular accidents [11].

Platelets constitute one of the potential links between inflammation, psoriasis, and elevated cardiovascular risk. Platelet reactivity in patients with psoriasis is elevated compared to healthy controls and correlates with the severity of the disease [12,13,14,15,16]. On the other hand, an insufficient response to antiplatelet treatment in patients with coronary artery disease is a renowned risk factor of myocardial infarction, increased mortality, and stent thrombosis, a life-threatening complication of percutaneous coronary intervention [17,18,19,20]. Moreover, excessive platelet reactivity is a predictor of cardiovascular and all-cause mortality, and a risk equivalent to the presence of coronary artery disease in patients not receiving antiaggregatory therapy as well [21]. Finally, elevated concentrations of inflammatory markers, such as C-reactive protein (CRP), tumor necrosis factor α (TNF-α), interleukin 6 (IL-6), or IL-10, correlate to higher platelet reactivity [21,22,23,24,25,26].

Taking into account the complexity of the potential interplay between inflammation, platelet function, and psoriasis, we attempted to investigate the determinants of platelet activation in patients with psoriasis.

## 2. Materials and Methods

### 2.1. Study Design

This study was a single-center, investigator-initiated, prospective, observational trial that aimed to evaluate the clinical factors affecting platelet reactivity in patients hospitalized for an exacerbation of psoriasis. The trial was conducted in accordance with the principles contained in the Declaration of Helsinki and Good Clinical Practice guidelines. The study was approved by the Ethics Committee of Nicolaus Copernicus University in Toruń, Collegium Medicum, in Bydgoszcz, Poland. All study participants provided written informed consent.

In the current trial, exacerbation of the disease was defined as a worsening of symptoms requiring hospitalization according to the attending physician. To be included in the study patients had to be diagnosed with psoriasis at least 6 months before their enrolment and between 18 and 80 years old. Exclusion criteria included (1) diagnosis of a psoriasis type other than psoriasis vulgaris; (2) history of generalized inflammatory disease other than psoriatic arthritis; (3) severe liver disease; (4) glomerular filtration rate below 30 mL/min/1.73 m^2^; (5) use of antiplatelet agents (aspirin, ticlopidine, clopidogrel, prasugrel, or ticagrelor) within 14 days of enrolment; (6) chronic use of low-molecular-weight heparin or oral anticoagulation; (7) use of nonsteroidal anti-inflammatory drugs within 3 days of enrolment; (8) use of phototherapy, cyclosporin A, methotrexate, or acitretin within 1 month of enrolment; (9) use of biological treatment within 2 months of enrolment; (10) platelet count below <120,000/mm^3^; and (11) pregnancy.

Succeeding patients with an exacerbation of psoriasis were admitted to the study site (Department of Dermatology, Venerology and Immunodermatology, Dr. A. Jurasz University Hospital, Bydgoszcz, Poland) and were screened for eligibility. Enrolled patients underwent standard dermatological assessment and single platelet function testing with light transmission aggregometry (LTA), which was performed on the first morning of hospitalization.

### 2.2. Dermatological Evaluation

Upon hospital admission, study participants underwent standard dermatological assessment. Amongst others, all patients underwent evaluation of their body surface area (BSA), the Psoriasis Area and Severity Index (PASI), and the Dermatology Life Quality Index (DLQI), which are widely recognized and employed [27,28]. Briefly, their BSA was estimated using the “rule of nines”, where involvement of the head and neck, each arm, anterior and posterior leg, and the four trunk quadrants, respectively, was awarded 9%, leaving 1% for the genitalia (the range of potential score: 0–100%) [28]. PASI score was obtained by entering the affected area and lesion characteristics into the dedicated formula (the range of potential scores: 0–72) [29]. Quality of life with the disease was evaluated with the use of the DLQI questionnaire containing ten questions regarding the impact of the disease on daily activities during the previous week (the range of potential overall scores: 0–30) [30].

### 2.3. Blood Sampling

Study participants were asked to remain fasting for at least 6 h prior to blood sampling. Additionally, they were requested to abstain from smoking for at least 30 min before drawing blood. Blood samples were obtained between 8 and 10 a.m., after at least 10 min of rest, using a 21G cannula introduced into one of the forearm veins. The initial 5 mL of blood was discarded in order to prevent the spontaneous activation of thrombocytes. Samples were drawn into vacuum tubes with sodium citrate 3.2% (Vacuette^®^, Greiner Bio-One GmbH, Leipzig, Germany), which were then gently stirred five times to ensure the appropriate mixing of blood and the anticoagulant.

### 2.4. Platelet Function Assessment

Platelet reactivity was evaluated with LTA using the Optical Aggregation System Chrono-log^®^ 490-2D (Chrono-log Corporation, Havertown, PA, USA). Assessment of platelet reactivity was performed using 5 μM adenosine diphosphate (ADP). The protocol for platelet aggregation assessment was based on recommendations for LTA standardization issued by the Working Party from the Platelet Physiology Subcommittee of the Scientific and Standardization Committee of the International Society on Thrombosis and Haemostasis, and North American consensus guidelines for medical laboratories that perform platelet function testing using LTA [31,32]. All measurements were performed within 2 h of blood sampling.

Platelet reactivity was expressed as maximal platelet aggregation (MPA), which quantifies the degree of the maximal activation of thrombocytes after a platelet agonist is added to the examined sample [31,32]. In line with the previously established threshold for high platelet reactivity (HPR), this was diagnosed in cases where the MPA was greater than 46% [33].

### 2.5. Sample Size Calculation

A pilot study was performed in order to estimate the final sample size. Based on the results obtained from the analysis of the first 25 enrolled patients, we decided to evaluate the sample size based on the correlation between MPA and the platelet count. Assuming a two-sided alpha value of 0.05, we calculated that, for the correlation coefficient, the enrolment of 105 patients was necessary to provide 85% power to demonstrate a significant correlation between the chosen variables. The inclusion of an additional 15 patients was planned to ensure adequate analysis in case of drop outs or missing data.

### 2.6. Statistical Analyses

Statistical calculations were performed using the Statistica 13 package (StatSoft, Tulsa, OK, USA) and R version 3.5.0 (The R Foundation, Vienna, Austria). Data for the duration of the disease, time from previous exacerbation, DLQI, BSA, and PASI, were presented as means with standard deviations. Data for age, BMI, CRP, platelet count, MPV, fasting glucose, total cholesterol, high-density lipoprotein-cholesterol, low-density lipoprotein-cholesterol, triglycerides, and MPA were presented as medians and interquartile ranges. Selected continuous data were transformed into dichotomous variables according to chosen thresholds (BMI < 25 vs. ≥25 kg/m^2^; BSA < 10 vs. ≥10%; PASI < 10 vs. ≥10 points; MPV ≤ 10.5 vs. >10.5 fL). Comparisons between dichotomous variables were performed by a χ^2^ test with Pearson correction. Correlations between MPA and selected variables were tested with the use of Pearson’s coefficient and a correlation coefficient significance test. Mixed models with random effects were applied in order to evaluate the impact of selected clinical and laboratory variables on MPA. The maximum likelihood method was used to estimate variance. Coefficients of determination (R^2^) were also provided to indicate how well data fit a statistical model. Linear mixed models were created for twenty-two variables. Next, a two-step analysis based on mixed models with random effects was performed, and variables were removed from further steps of analysis in cases where *p* > 0.05. In all analyses *p* < 0.05 was considered significant.

## 3. Results

The study population included 120 patients with psoriasis, with a median age of 38.0 [30.5–54.0] years old, of which 34% were female (Table 1). None of the study participants had a history of coronary artery disease, stroke, transient ischemic attack, peripheral artery disease, or any other condition requiring chronic antiplatelet therapy. The median MPA was 75%, and as many as 109 patients (90.8%) had HPR (i.e., MPA > 46%).

**Figure 1:**
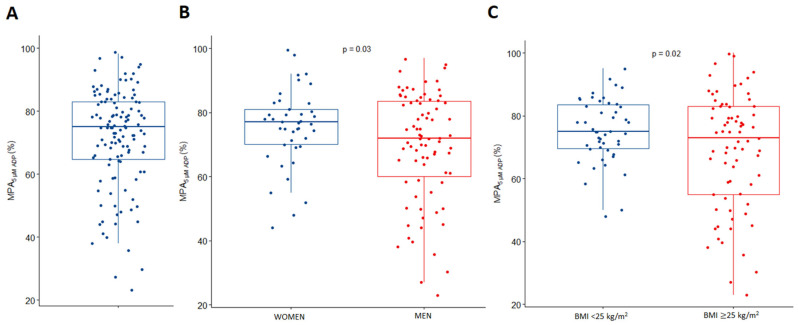
Platelet reactivity of study participants. (**A**) all study participants; (**B**) comparison between women and men; (**C**) comparison between patients without and with elevated BMI. Comparisons between groups presented in (**B**,**C**) were made with the use of χ^2^ test with Pearson correction. ADP: adenosine diphosphate; BMI: body mass index; MPA: maximal platelet aggregation.

Among the compared subgroups, women had a higher MPA than men (77% vs. 72%; *p* = 0.03), and those with BMIs < 25 kg/m^2^ showed higher platelet reactivity compared to patients with BMIs ≥ 25 kg/m^2^ (75% vs. 73%; *p* = 0.02) (Figure 1). There was also a trend towards higher MPA in patients with psoriatic arthritis compared to those without joint involvement, but the difference between the groups did not reach statistical significance (82% vs. 74%; *p* > 0.05) (Table 2).

**Figure 2:**
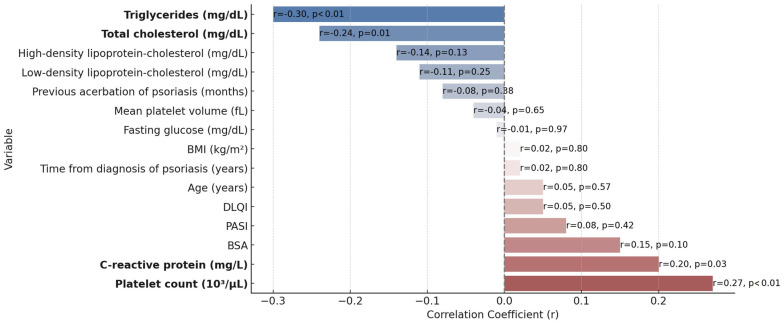
Correlations between clinical and laboratory variables and maximal platelet reactivity in patients with psoriasis. Evaluation of correlations between MPA and presented variables was performed with the use of Pearson’s coefficient and a correlation coefficient significance test. Variables with statistically significant correlation coefficients are marked in bold. BMI: body mass index; BSA: body surface area; DLQI: Dermatology Life Quality Index; MPA: maximal platelet aggregation; PASI: Psoriasis Area and Severity Index.

The correlation between platelet reactivity and 15 different clinical and laboratory variables was evaluated (Figure 2, Table 3). A positive correlation was observed between MPA and the platelet count (r = 0.27; *p* < 0.01), as well as the CRP concentration (r = 0.20; *p* = 0.03). On the other hand, a negative correlation was observed for total cholesterol (r = −0.24; *p* = 0.01) and triglycerides (r = −0.30; *p* < 0.01).

Next, 22 variables were evaluated in linear mixed models with random effects (Table 4). According to this analysis, four of these variables had an impact on platelet reactivity. MPA increased by 0.2% with an increase in the CRP concentration by 1 mg/L (R^2^ = 0.04; *p* = 0.03), and by 0.07% with each additional 10^3^/μL rise in the platelet count (R^2^ = 0.07; *p* < 0.01). Conversely, MPA decreased by 0.09% and 0.05% with each additional mg/dL of total cholesterol (R^2^ = 0.05; *p* = 0.01) and triglycerides (R^2^ = 0.07; *p* < 0.01), respectively. Subsequently, a multidimensional model with random effects that included the abovementioned four variables was created (Table 5). In the first step of this analysis, the impact on platelet reactivity was verified only for the platelet count and triglycerides, which was later confirmed in a further model that included only these two variables—MPA increased by 0.07% with each additional 10^3^/μL of platelets (R^2^ = 0.07; adjusted *p* < 0.01) and decreased by 0.05% with each increase in triglycerides by 1 mg/dL (R^2^ = 0.07; adjusted *p* < 0.01).

## 4. Discussion

The current study aimed to contribute to our understanding of the pathophysiology of psoriasis and its potential implications on platelet reactivity in patients during an exacerbation of the disease. Several previously published studies reported higher platelet aggregation in patients with psoriasis compared to healthy subjects [12,13,14,15,16], thus this trial was planned as a single-arm study without healthy controls.

Our initial data analysis plan aimed to explore the relationships between various clinical features and platelet reactivity, both in terms of crude values and as a percentage of patients exhibiting HPR. However, it is noteworthy that a substantial proportion (91%) of our study participants demonstrated HPR. This high prevalence prevented the statistical evaluation of factors predisposing a person to HPR, due to the limited number of patients without elevated platelet aggregation.

To put this into perspective, HPR is found upon hospital admission in 76–100% of patients with acute myocardial infarction, a potentially fatal condition directly caused and related to uncontrolled platelet hyperreactivity [34]. As previously mentioned, the presence of HPR in patients with acute coronary syndrome increases the risk of unfavorable thrombotic complications, including increased cardiovascular mortality [17,18,19,20]. Recently, Berger et al. reported that ADP-induced HPR in patients with angiographically excluded coronary artery disease who were not receiving antiplatelet agents (other than aspirin) is related to a 40% increase in cardiovascular mortality compared to patients without HPR (hazard ratio 1.2, 95% confidence interval 1.1 to 1.9; *p* = 0.0175). Interestingly, in their 8.4-year follow-up occurrence of cardiovascular death in this group was comparable with patients without altered platelet reactivity, but with confirmed coronary artery disease, which demonstrates the significance of platelet function abnormalities for cardiovascular prognosis [21]. Currently, there are no available data on the impact of HPR on the long-term prognosis of patients with psoriasis.

In this trial, we did not identify a significant association between platelet reactivity and variables such as BSA, PASI, or DLQI. It is worth noting that there are existing reports in the literature that describe relationships between platelet function and the severity of psoriasis. Chandrashekar et al. reported a strong positive correlation between PASI and ADP-induced platelet aggregation (r = 0.71; *p* < 0.0001). A similar relationship was shown for mean platelet volume (r = 0.83; *p* < 0.0001), soluble P-selectin (r = 0.66; *p* < 0.0001), CRP (r = 0.36; *p* = 0.004), and IL-6 (r = 0.408; *p* = 0.001) [15]. Garbaraviciene et al. described the normalization of elevated thrombocyte aggregation in cases of the successful treatment of psoriasis, reporting a strong positive correlation between platelet P-selectin and PASI (r = 0.5; *p* < 0.000001), as well as between the change in the PASI and the change in P-selectin expression (r = 0.4; *p* = 0.006) [16]. In line with this, Tamagawa-Mineoka et al. also depicted a positive correlation between platelet factor 4, an alternative platelet activity marker, and PASI (r = 0.66; *p* = 0.01) [14].

When we analyzed the MPA in predefined subgroups, significant differences were present only in two compared pairs. In the current study, women hospitalized for an exacerbation of psoriasis presented with higher MPA than men (77% vs. 72%; *p* = 0.03). Such a relationship has never been described before in psoriatic patients; however, higher platelet reactivity in women, or the trend towards it, has been previously reported in other clinical scenarios. Still, the absolute magnitude of the differences between genders observed in other trials was reasonably small and most likely without clinical significance [35,36,37].

Among our study population, in direct comparison, psoriatic patients without elevated BMIs (<25 kg/m^2^) had higher MPA than those with elevated BMIs (BMI ≥ 25 kg/m^2^). The absolute difference in MPA between these groups was only 2%, but it reached statistical significance (75% vs. 73%; *p* = 0.02). We found this result surprising, as we rather anticipated the opposite, as obesity causes the chronic low-grade inflammation of adipose tissue [38], and interactions between inflammation and elevated platelet aggregation have been previously described [21,22,23,24,25,26]. Indeed, our finding is inconsistent with other reports. In overweight or obese healthy controls, or patients with coronary artery disease, platelet aggregation in response to different platelet agonists (arachidonic acid, thrombin, collagen, ADP) is generally greater than in patients with no elevated BMI [39,40,41]. Notably, the relationship between platelet reactivity and BMI was not confirmed in the linear or multidimensional models we have applied in our study.

In our trial, the highest mean MPA was seen in patients with psoriatic arthritis. However, when compared with patients without psoriatic arthritis, the difference in platelet reactivity was not significant, although a clear trend was visible (82% vs. 74%; *p* > 0.05). Importantly, only 10% (*n* = 12) of our study participants had psoriatic arthritis; therefore, the lack of statistical significance might have resulted from an insufficient number of patients with joint involvement. Di Minno et al. not only showed that patients with psoriatic arthritis demonstrate higher MPA and HPR rates than healthy controls, but also that patients with minimal disease activity during TNF-α inhibitor treatment exhibit comparable platelet aggregation to that seen in controls. In their study, subjects with active disease were diagnosed with HPR more often than those achieving minimal disease activity. Last but not least, the authors reported an increase in the CRP concentration and a reduction in the clinical improvement of the TNF-α inhibitor in correlation with the rise of MPA [42]. These results suggest the presence of a complicated interplay between psoriatic arthritis, inflammation, and platelet hyperreactivity, similar to what is observed in plaque psoriasis.

In further steps of statistical analysis, we found a positive correlation between MPA and the platelet count, as well as the CRP concentration, while a negative correlation was observed for total cholesterol and triglycerides. Next, an increase in platelet activity secondary to an increase in the platelet count and a reduction in platelet aggregation due to an increase in triglycerides was confirmed in the mixed models with random effects. The positive relationship of platelet reactivity with the CRP and the negative with total cholesterol were significant only according to the linear mixed models with random effects, and were not corroborated by multidimensional models with random effects. The impact of these variables on platelet aggregation in our study was moderate at most. The values of the statistically significant correlation coefficients varied between −0.30 and 0.27 (Table 3), and R^2^ values for the applied models with random effects did not exceed 0.07 (Table 4 and Table 5), indicating that changes in platelet reactivity in psoriasis can be explained only partially by the results we have described.

Data on the influence of the platelet count on platelet reactivity are sparse and mainly originating from patients with coronary artery disease treated with antiaggregatory therapy, but are in line with our findings. In patients with acute coronary syndromes, the odds of HPR during the initial hours of antiplatelet treatment increase proportionally to the increase in the platelet count [43]. In thienopyridine-treated patients who undergo elective coronary stenting, the immature platelet count was the strongest predictor of in-treatment platelet reactivity [44]. The clinical significance of this interaction in psoriatic patients needs further elucidation in a properly sized trial with an appropriate follow-up.

CRP is one of the most commonly used markers of inflammation. It has been shown to correspond to the severity of psoriasis [45,46]. In our study, it was positively correlated with a higher platelet reactivity in patients with an exacerbation of psoriasis. Chandrashekar et al. previously described the same relationship, but in their trial the correlation between ADP-induced platelet aggregation and CRP was 3-fold greater than what we observed in our population (r = 0.63; *p* < 0.0001). Interestingly, in that study, an even stronger positive correlation was found for IL-6 (r = 0.80; *p* < 0.0001) [15]. Similarly, in patients who recently underwent acute coronary syndrome, a rise of their CRP concertation above 1 mg/L was related to higher rates of HPR (CRP > 1 mg/L vs. ≤1 mg/L: 49.1% vs. 36.4%; *p* = 0.012) [47]. Generally, increased levels of inflammatory markers (CRP, TNF-α, IL-6, IL-10) correspond to higher platelet reactivity [21,22,23,24,25,26]. These findings suggest the presence of a potential connection between an elevated inflammatory state and platelet hyperactivity, which both were reported to increase the risk of adverse cardiovascular events seen in psoriatic patients and affect their prognosis [48].

According to the rule “the lower, the better”, different lipid fractions constitute therapeutic targets in both the primary and secondary prevention of cardiovascular events [9]. Psoriasis is typically associated with various lipid disorders, including hypertriglyceridemia and hypercholesterolemia [49,50]. The current study demonstrated an inverse relationship between the concentration of triglycerides and platelet reactivity. An identical connection was shown for total cholesterol. Similar observations were made in a large-scale analysis including 3416 subjects, where an inverse association between ADP-induced platelet aggregation and the total cholesterol level was described. The same trial indicated a relationship between altered platelet activity and the concentration of triglycerides [51]. On the other hand, lipid-lowering drugs generally tend to reduce platelet activity [52]. Thus, our results must be interpreted with caution due to some inconsistencies in the available data, as well as the unknown mechanisms underlying these potential interactions. In particular, the presence of an inverse correlation between lipid fractions and platelet aggregation could be considered controversial. One could conclude that an increase in triglycerides and total cholesterol concentrations might be beneficial for patients with psoriasis due to the mitigation of platelet hyperreactivity. Nevertheless, it should be stressed that lipid-lowering therapy is crucial for the management of cardiovascular risk, and our purely mechanistic observations do not substantiate the evading of dyslipidemia therapy in patients with psoriasis.

We believe that further, more complex studies are necessary to put our findings into a clinical perspective. Such trials should evaluate the platelet reactivity in psoriatic patients at various timepoints (e.g., baseline, during exacerbation, post treatment, etc.) to identify individuals with chronic platelet hyperreactivity and to determine the risk factors for HPR. These observations should be followed up in a long-term and properly sized study to verify whether a connection between excessive platelet reactivity and worse cardiovascular outcomes exists in patients with psoriasis. While this is purely speculative, observing such a relationship could provide the rationale for the clinical evaluation of antiplatelet agents in preventing adverse cardiovascular events in psoriasis patients.

## 5. Study Limitations

Our research had several limitations that require mentioning. First, platelet function was assessed using only one method, and only once, on the first morning of hospitalization, without follow-up after treatment. Furthermore, CRP was the only inflammatory marker that was evaluated, as a routine assessment of IL-6 or TNF-α was not available at our institution at the time of study conduction. Because it was an observational study, the decision regarding the need for hospitalization for psoriasis exacerbation was left to the physician’s discretion, and no additional trial-specific criteria were used, which might have increased the population’s heterogeneity. Finally, it must be underlined that while we have identified certain associations between platelet reactivity and clinical/laboratory features in psoriasis patients, there may be additional factors not investigated in this study that could influence platelet reactivity.

## 6. Conclusions

The increased platelet reactivity observed in patients with psoriasis appears to be multifactorial and related to several clinical and laboratory features. Further research is warranted to put these findings into a clinical perspective.

## Figures and Tables

**Table 1:** Baseline characteristics of trial participants.

Variable	Patients with Psoriasis (*n* = 120)
MPA (%)	75% [65–83%]
HPR	109 (90.8%)
Age (years)	38.0 [30.5–54.0]
Women	41 (34.2%)
BMI (kg/m^2^)	26.5 [24.1–29.6]
Time from diagnosis of psoriasis (years)	18.0 ± 10.9
Previous exacerbation of psoriasis (months)	3.2 ± 2.5
Psoriatic arthritis	12 (10.0%)
DLQI (points)	15.2 ± 6.2
BSA (%)	22.4 ± 17.1
PASI (points)	13.1 ± 7.8
Metabolic syndrome	34 (28.3%)
Hypertension	21 (17.5%)
Diabetes mellitus	4 (3.3%)
Asthma	3 (2.5%)
Current smoking	58 (48.3%)
C-reactive protein (mg/L)	2.48 [0.83–5.68]
Platelet count (10^3^/μL)	257 [227–300]
Mean platelet volume (fL)	10.4 [10.0–11.0]
Fasting glucose (mg/dL)	88 [82–93]
Total cholesterol (mg/dL)	206.5 [174.5–225.0]
High-density lipoprotein-cholesterol (mg/dL)	49.5 [44.0–57.5]
Low-density lipoprotein-cholesterol (mg/dL)	130.0 [108.0–146.0]
Triglycerides (mg/dL)	115.0 [75.5–158.0]
Topical treatment	119 (99.2%)
History of methotrexate use	46 (38.3%)
History of cyclosporin use	30 (25.0%)
History of acitretin use	16 (13.3%)
History of UVA treatment	1 (0.83%)
History of UVB311 treatment	29 (24.2%)
History of PUVA treatment	38 (31.7%)
History of biological treatment	19 (15.8%)
Family history of psoriasis	59 (49.2%)
Family history of coronary artery disease	51 (42.5%)

Data are shown as mean ± SD, median [interquartile range], or number (%). BMI: body mass index; BSA: body surface area; DLQI: Dermatology Life Quality Index; HPR: high platelet reactivity; MPA: maximal platelet aggregation; PASI: Psoriasis Area and Severity Index; PUVA: psoralen and ultraviolet A; UVA: ultraviolet A; UVB311: ultraviolet B, 311 nm.

**Table 2:** Comparison of platelet reactivity between selected subgroups of patients.

Compared Groups	MPA (%)	*p*
Women vs. men	77 [70–82] vs. 72 [59–84]	0.03
BMI < 25 kg/m^2^ vs. ≥25 kg/m^2^	75 [69–84] vs. 73 [55–83]	0.02
BSA < 10% vs. ≥10%	74 [69–79] vs. 75 [61–84]	0.33
PASI < 10 points vs. ≥10 points	75 [67–84] vs. 73 [56–82]	0.18
Psoriatic arthritis vs. no psoriatic arthritis	82 [72–88] vs. 74 [64–83]	>0.05
Smokers vs. non-smokers	74 [66–82] vs. 75 [63–83]	0.94
Hypertension vs. no hypertension	77 [67–84] vs. 74 [64–83]	0.2
Mean platelet volume ≤ 10.5 fL vs. >10.5 fL	74 [63–84] vs. 75 [65–83]	0.89
Metabolic syndrome vs. no metabolic syndrome	78 [66–84] vs. 74 [64–83]	0.27

Data are shown as median [interquartile range]. Comparisons between groups were made with the use of χ^2^ test with Pearson correction. BMI: body mass index; BSA: body surface area; MPA: maximal platelet aggregation; PASI: Psoriasis Area and Severity Index.

**Table 3:** Correlations between clinical and laboratory variables and platelet reactivity.

Variable	r	*p*
Age (years)	0.05	0.57
BMI (kg/m^2^)	0.02	0.8
Time from diagnosis of psoriasis (years)	0.02	0.8
Previous exacerbation of psoriasis (months)	−0.08	0.38
DLQI	0.05	0.5
BSA	0.15	0.1
PASI	0.08	0.42
C-reactive protein (mg/L)	0.2	0.03
Platelet count (10^3^/μL)	0.27	<0.01
Mean platelet volume (fL)	−0.04	0.65
Fasting glucose (mg/dL)	−0.01	0.97
Total cholesterol (mg/dL)	−0.24	0.01
High-density lipoprotein-cholesterol (mg/dL)	−0.14	0.13
Low-density lipoprotein-cholesterol (mg/dL)	−0.11	0.25
Triglycerides (mg/dL)	−0.3	<0.01

Evaluation of correlations between MPA and presented variables was performed with the use of Pearson’s coefficient and correlation coefficient significance test. BMI: body mass index; BSA: body surface area; DLQI: Dermatology Life Quality Index; MPA: maximal platelet aggregation; PASI: Psoriasis Area and Severity Index.

**Table 4:** Influence of various clinical variables on platelet reactivity according to linear mixed models with random effects.

Variable	Value	SE	*p*	R^2^
Age (years)	0.07	0.11	0.54	<0.01
Women	5.89	3.02	>0.05	0.03
BMI (kg/m^2^)	0.09	0.32	0.77	<0.01
Time from diagnosis of psoriasis (years)	0.03	0.13	0.83	<0.01
Previous exacerbation of psoriasis (months)	−1.36	3.31	0.68	<0.01
Psoriatic arthritis	8.68	4.78	0.07	0.03
DLQI (points)	0.14	0.24	0.55	<0.01
BSA (%)	0.14	0.08	0.1	0.02
PASI (points)	0.16	0.19	0.39	0.01
Metabolic syndrome	3.2	3.21	0.32	0.01
Hypertension	4.43	3.81	0.25	0.01
Diabetes mellitus	3.94	8.09	0.63	<0.01
Asthma	13.22	9.24	0.16	0.02
Current smoking	−0.22	2.91	0.94	<0.01
C-reactive protein (mg/L)	0.2	0.09	0.03	0.04
Platelet count (10^3^/μL)	0.07	0.02	<0.01	0.07
Mean platelet volume (fL)	−0.48	1.26	0.71	<0.01
Fasting glucose (mg/dL)	0	0.09	0.97	<0.01
Total cholesterol (mg/dL)	−0.09	0.04	0.01	0.05
High-density lipoprotein-cholesterol (mg/dL)	−0.17	0.11	0.13	0.02
Low-density lipoprotein-cholesterol (mg/dL)	−0.05	0.04	0.26	0.01
Triglycerides (mg/dL)	−0.05	0.02	<0.01	0.07

Mixed models with random effects were applied in order to evaluate the impact of selected variables on MPA. The maximum likelihood method was used to estimate variance. Coefficients of determination (R^2^) were also provided to indicate how well data fit a statistical model. BMI: body mass index; BSA: body surface area; DLQI: Dermatology Life Quality Index; MPA: maximal platelet aggregation; PASI: Psoriasis Area and Severity Index; SE: standard error.

**Table 5:** Influence of various clinical variables on platelet reactivity according to multidimensional models with random effects.

Variable	Value	SE	Adjusted *p*	R^2^
The first step				
C-reactive protein (mg/L)	0.12	0.09	0.21	0.01
Platelet count (10^3^/μL)	0.06	0.02	0.01	0.05
Total cholesterol (mg/dL)	−0.05	0.04	0.17	0.02
Triglycerides (mg/dL)	−0.04	0.02	0.04	0.04
The second step				
Platelets (10^3^/μL)	0.07	0.02	<0.01	0.07
Triglycerides (mg/dL)	−0.05	0.02	<0.01	0.07

A two-step analysis based on mixed models with random effects was performed to assess the impact of selected variables on MPA. Variables were removed from further steps of the analysis in cases where *p* > 0.05. SE: standard error.

## Data Availability

The data sets used and analyzed during the current study are available from the corresponding author on reasonable request.
